# Exploring Surface Acoustic Waves (SAWs) for Water Quality Sensor’s Anti-Biofouling Application: A New Direction for Acoustic Waves

**DOI:** 10.3390/s26113480

**Published:** 2026-06-01

**Authors:** Asma Akther, Tim Malthus, Anusuya Willis, Régine Chantler, Stephen Gensemer, Hendrik Falk, Hanne Stang, Charlottle Farnworth, Anu Kumar

**Affiliations:** Commonwealth Scientific and Industrial Research Organisation (CSIRO), Melbourne, VIC 3168, Australia; tim.malthus@csiro.au (T.M.);

**Keywords:** anti-biofouling, algae, vibration, surface acoustic waves (SAWs), sensors, sound propagation acoustic field, vibration, water quality monitoring, inland water

## Abstract

Biofouling presents numerous challenges across various sectors, including aquaculture, agriculture, infrastructure, and medicine. The development of anti-biofouling techniques remains a significant challenge. In the water industry, biofouling on monitoring sensors substantially compromises the accuracy of measurements by interfering with the sensors’ measuring ability. Biofouling also significantly increases the running costs by increasing the frequency of maintenance needed to keep sensors clean and accurate. Consequently, anti-biofouling techniques are widely employed to clean in situ optical sensors, ensuring accurate measurements while minimizing overall system costs. The conventional approach for preventing biofouling from in situ sensors typically involves the application of coatings, mechanical brushes, ultraviolet radiation, and ultrasonic waves, which possess distinct advantages and disadvantages contingent upon their application. The challenges associated with protecting the small windows of water quality sensors from biofouling over extended periods using current methods are either expensive or adversely affect the integrity of monitoring data. This study introduces a low-cost centimeter-scale high-frequency surface acoustic wave (SAW) device to protect the small windows of in situ water quality sensors continuously from biofouling, functioning as an auxiliary anti-biofouling mechanism. This study found that this 16 MHz SAW device can mitigate the formation of biofilms by adhesive diatom strains CS-1664, CS-1665, and by planktonic algae CS-327 by approximately 98% in comparison to control conditions, functioning effectively as an anti-biofouling tool for itself and surrounding surfaces without adversely affecting aquatic organisms. The dimension and resonance frequency (RF) of the SAW device are also capable of being fabricated according to the area requiring cleaning. A miniaturized 16 MHz SAW device can sustain operation for prolonged periods up to a couple of months without maintenance, at a low cost and power consumption, providing a new anti-biofouling technology. This methodology aims to assist the Australian inland and coastal water quality monitoring system by reducing maintenance costs while simultaneously enhancing the longevity of sensors submerged in water for extended periods.

## 1. Introduction

**Figure 1:**
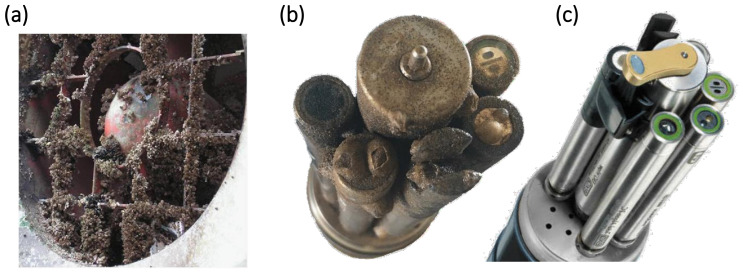
Examples of biofouling on submerged equipment depicted as: (**a**) the vessel hull image was adapted from M. Legg [7]; and (**b**,**c**) after and before images of in situ multiprobe water quality sensors after long-term deployment in fresh water.

Microbial and macrobial biomass attachment on devices is a universal phenomenon responsible for biofouling, where the accumulation and growth of these organisms cause a significant problem for different industries [1,2]. Generally, microorganisms, such as bacteria, algae, and protozoa, create a mucilaginous biofilm that negatively affects the functioning of equipment [3,4]. The biofilm layer also causes issues for ships and boats; for example, when the biofilm forms on the hull, increasing the hydrodynamic drag forces and thus increases maintenance costs and fuel consumption [5,6]. In this study, we focus on inland and coastal water monitoring systems, where biofouling is a significant challenge for accurate water quality monitoring. In inland sensor systems, biofilms develop through the sequential attachment of bacteria and algae that secrete extrapolymeric substances (EPSs) or mucilage that creates a habitat for the algae and forms a layer on submerged surfaces. Some examples are shown in Figure 1 of a vessel and an optical sensor where thick biofilms are attached.

Microorganisms commonly adhere to and proliferate on solid surfaces submerged in water. On submerged sensors, this unwanted biofilm hinders the accuracy of water quality assessments. The primary objective of anti-biofouling research is to develop materials or equipment that are antifouling while being environmentally low-impact, biocompatible, and sustainable, i.e., without negatively affecting the ecosystem. There are various chemical, biological, and physical methods available to prevent biofouling [8,9]. A summary of some popular techniques, including their mechanisms, applications, advantages, and disadvantages, is provided in Table 1. One of the methods of mitigating biofouling involves applying a specific coating, often containing toxic substances, to the surface, thereby protecting it from microbial growth for a specific duration [10]. This represents a traditional approach to safeguarding underwater devices from biofouling incidents. However, there are challenges associated with this method, including potential toxicity and threats to aquatic life [5,11]. In the case of the use of optical sensors, the extra layer of anti-biofouling coating can interfere with the optical signals, ultimately affecting the results. Another common method involves the use of mechanical brushes or wipers to physically clean optical windows by being positioned adjacent to the windows and performing periodic cleaning. However, water measurements cannot be conducted while the brushes are positioned in front of the window, and the system requires substantial power to maintain cleanliness.

**Figure 2:**
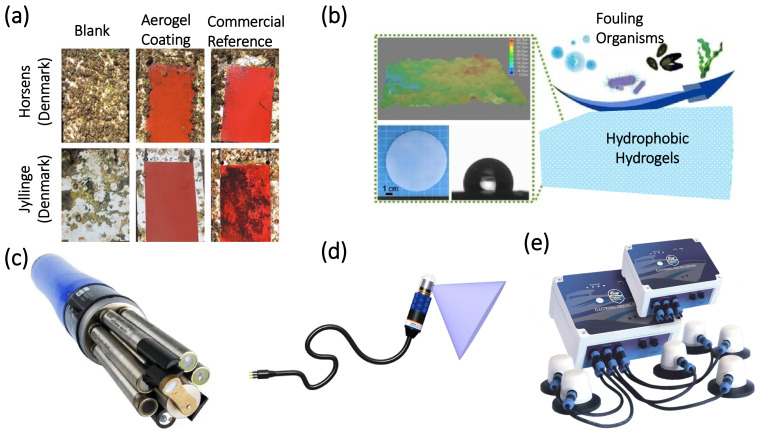
Different methods for preventing biofouling on various surfaces are shown. (**a**) Static biocide-releasing coating [45]. (**b**) Anti-adhesive hydrophobic hydrogel [46]. (**c**) Standalone and built-in anti-biofouling wiper [47]. (**d**) The UV-C irradiated panel is designed to protect surfaces from anti-biofouling attacks [47]. (**e**) Ultrasonic waves-based antibiofouling protection for ships. This image was adapted from the Electronic Fouling Control (EFC) website.

Each of the methods mentioned above offers specific advantages and limitations depending on the application. Some commonly used anti-biofouling technologies are illustrated in Figure 2. In this study, we focus on developing an anti-biofouling device designed specifically for the optical windows of in situ water quality sensors, with the goal of preventing biofouling on equipment in inland aquatic environments over long periods. The objective is to lower total maintenance and operating costs while maintaining the accuracy and reliability of water quality measurements. To address these challenges, our approach uses a miniaturized, tunable, high-frequency SAW device as an add-on component. This compact system effectively prevents and removes biofouling from the sensor optical window without harming the surrounding ecosystem or interfering with data collection.

A SAW is a mechanical wave that propagates along piezoelectric substrates through electromechanical coupling and has been widely exploited for applications such as particle trapping, micro-centrifugation, and particle separation [48]. SAW-based platforms are most commonly associated with microfluidic manipulation and consequently have found significant utility in point-of-care diagnostic systems [49,50]. In contrast to these established applications, the present study investigates the use of a conventional 16 MHz SAW device as an anti-biofouling mechanism for optical in situ water-quality sensors. In the context of mitigating biofouling on optical surfaces, SAW-generated acoustic radiation forces interact with microorganisms in a manner analogous to a mechanical sweeping action, displacing and aggregating them away from the sensing region. Capitalizing on the compact form factor, low mass, and tunable power consumption inherent to microelectromechanical systems (MEMS), this approach offers a promising pathway for enhancing long-term deployment of in situ water-quality monitoring systems by reducing fouling-induced maintenance requirements.

This work introduces a novel, tunable SAW-based approach capable of preventing biofouling accumulation with low power consumption while operating as a fully contactless system that does not interfere with water quality measurements. This study also investigates the ability of high-frequency SAW excitation to disrupt early-stage biofilm development. The methodology focuses on the fabrication and characterization of centimeter-scale, custom high-frequency SAW devices designed to inhibit microalgal settlement and adhesion on optical surfaces, thereby suppressing the initiation and progression of biofilm formation.

## 2. Materials and Methods

### 2.1. SAW Device

**Figure 3:**
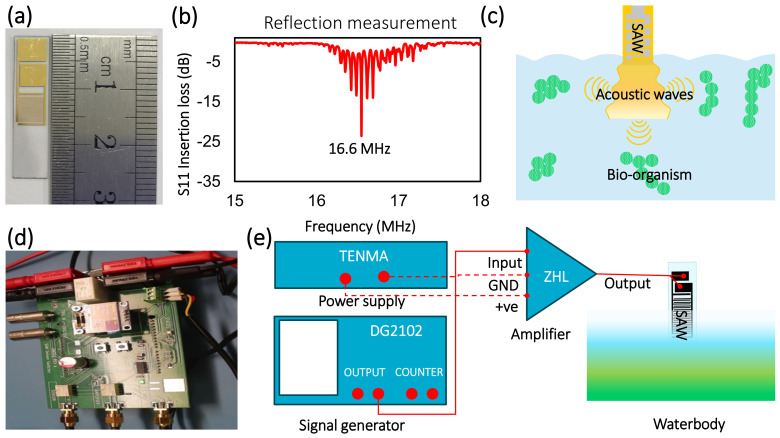
(**a**) A 16 MHz fabricated SAW device designed to prevent algae and other bio-organisms from adhering to surfaces for anti-biofouling applications. (**b**) Resonant Frequency (RF) response of the SAW device, showing a peak at 16.61 MHz as measured using a Vector Network Analyzer (VNA). (**c**) Schematic illustration of the anti-biofouling mechanism, where the SAW device generates acoustic energy that repels algae and microorganisms from the optical sensor surface. (**d**) Photograph of the customized printed circuit board (PCB) used to operate the SAW device. (**e**) Electrical connection diagram for the 16 MHz SAW anti-biofouling setup.

A piezoelectric substrate has undergone a standard photolithography process and wet etching to pattern inter-digital transducers (IDTs) onto its surface. The substrate utilized is a 0.5 mm-thick 128° YX-rotated single-crystal Lithium Niobate (LiNbO_3_), sourced from UniversityWafer Inc., Boston, USA. A subsequent deposition of 5 nm of chromium and 200 nm of gold was performed as part of the metallization procedures. Following the cleaning and dicing processes facilitated in a controlled cleanroom environment, SAW devices were fabricated with dimensions of 25.65 mm × 6.35 mm × 0.5 mm, which includes delay lines and a 4.2 mm aperture containing 20 pairs of IDTs. The wavelength of the device measures 234 μm, corresponding to a resonance frequency of 16.6 MHz, with a documented SAW phase speed for LiNbO_3_ of 3950 m/s, as depicted in Figure 3a,b [51]. A Vector Network Analyzer (VNA, Rigol, RSA3015N, 9 kHz–1.5 GHz, Sydney, Australia) was utilized to determine the device resonance frequency (RF) via connection through an RF SMA cable. Figure 3b illustrates one of the SAW device’s resonance frequencies, highlighting the specific frequency of 16.6 MHz for that device.

**Figure 4:**
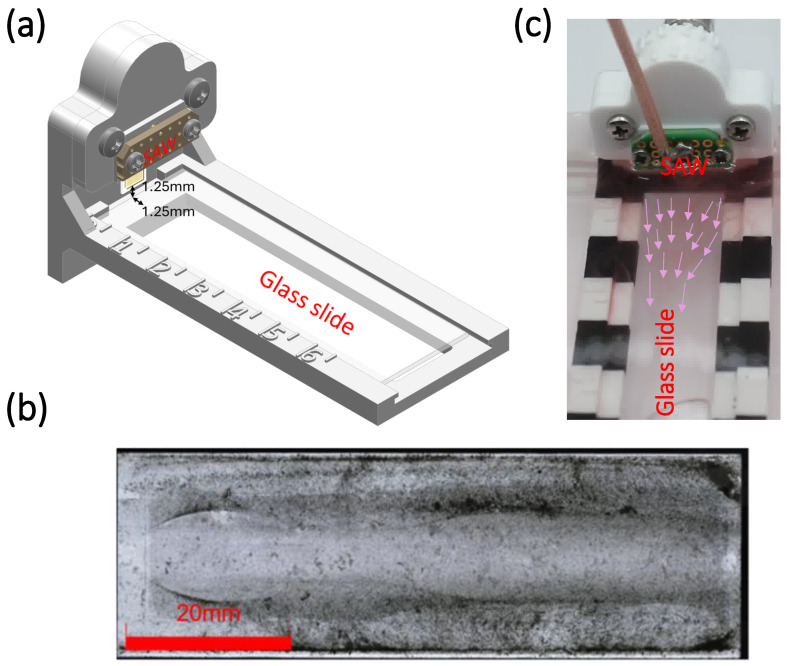
(**a**) The holder design of containing a SAW anti-biofouling device and a glass slide representing the optical window of an in situ sensor. The distance of a SAW device from the glass slide was determined to be 1.25 mm × 1.25 mm to effectively use SAW vibration to prevent algae growth on the surfaces. (**b**) A 3D electronic microscopy bright-field image of the complete glass slide showing the area of biofilm removed due to SAW-based acoustic vibration, and (**c**) image of a TSAW propagating vertically, which serves to displace the bio-organism from its active acoustic wave region. The pink arrows emphasize the propagation of micron-sized pink polystyrene particles, which represent algae, providing a color contrast that depicts the wave propagation toward the glass slide.

The mechanism of SAW production and the principle underlying the prevention of algal growth on a submerged surface are illustrated in Figure 3c. A 16 MHz SAW device, equipped with 20 pairs of inter-digital transducer (IDT) arrays, produces a sinusoidal wave propagation at its excitation frequency as a result of the substrate’s piezoelectric effect. The localized vibration generated by this device is referred to as “Travelling Surface Acoustic Waves (TSAW),” which propagate across the surface of the SAW device. This phenomenon offers a unique advantage for particle manipulation. The interplay of acoustic radiation forces, acoustic streaming forces, and surface acoustic vibrations inhibits the attachment of algae and bio-organisms to the surfaces [52]. During this process, the SAW transmits energy into the fluid flow, generating forces that are manipulable over distance [53] and producing a net force that drives micro-organisms away along the node and anti-node direction of the wave due to acoustic radiation forces, effectively detaching the algae from the surfaces, as demonstrated in Figure 3c. Conversely, acoustic streaming forces concentrate the algae outside the area of interest by introducing shear forces that hinder the early development of biofilm on the surfaces. Lastly, acoustic vibrations physically agitate the algae, reducing their ability to adhere to nearby surfaces, as shown in Figure 4c. The total acoustic force acting on microorganisms, such as bacteria or algae, in proximity to a SAW-active surface is the cumulative effect of acoustic radiation [54], streaming drag force [55], and vibration-induced inertial forces [56,57,58].

These effects can be conceptually described by expressing the total acoustic force acting on a microorganism near a SAW-active surface as the superposition of radiation, streaming drag, and inertial contributions:

(1) $$F_{\mathrm{total}}=-\nabla U+6\pi \mu av_{s}+m\omega^{2}A$$

where the Gor’kov potential *U* is given by:

(2) $$U=\frac{4\pi a^{3}}{3}\left(\frac{1}{2}f_{1}\kappa_{0}{\left|p\right|}^{2}-\frac{3}{4}f_{2}\rho_{0}{\left|v\right|}^{2}\right)$$

Here, *a* is the algal radius/dimension, μ is the dynamic viscosity of the fluid, $v_{s}$ is the acoustic streaming velocity, *m* is the particle mass, ω is the angular frequency of the SAW, and *A* is the displacement amplitude. The constants $\kappa_{0}$, $\rho_{0}$, *p*, v, $f_{1}$, and $f_{2}$ correspond to the fluid compressibility, fluid density, acoustic pressure, particle velocity, and material-dependent contrast factors, respectively [57,58]. This framework provides theoretical support for the experimentally observed detachment and displacement of algae under SAW excitation, reinforcing the use of TSAWs as a physical anti-biofouling mechanism.

### 2.2. Algae Preparation

All of the species were grown in WC+Si media at 20 °C under a 12:12 light-dark cycle with 20 μMol photons m^−2^ s^−1^ at the Australian National Algae Culture Collection (ANACC). The strains were cultivated for 7 days before the experiment. For the experiments, the SAW devices were placed in 1 L glass aquarium tanks with 400 mL of WC + Si medium [59]. Glass slides were attached to the device (Figure 4a). Some slides had algae attached (remove biofilm), while others had algae added once the SAW device was activated (prevention of biofouling growth). To conduct the adhesive diatoms and planktonic (removing biofilm), diatom cells were suspended by gentle agitation with a plastic pipette, and 3 mL was placed in a Petri dish containing the glass slides. The algae were allowed to settle for different periods, and then a 16 MHz SAW device was added to each tank for “With SAW” and “Without SAW” conditions. For the “preventing/protecting experiment with SAW,” a clean glass slide was placed in the 3D printer holder, a SAW device was activated, and 3 mL of suspended algae was released in the media above the glass slide surface via a plastic pipette. The 16 MHz SAW devices were installed in the tanks after a specific period to prevent both the surrounding surfaces from algae and biofilm attacks. The devices operated for extended periods, ranging from one week to a month [7], to assess their sustained cleaning capability at high power levels [60].

This study investigated a range of inland freshwater microalgae with differing adhesion characteristics and exposure durations to evaluate the effectiveness of a 16 MHz SAW device as a contactless add-on anti-biofouling solution for in situ water-quality sensor windows. Table 2 summaries the experimental conditions across three treatments: control or “Without SAW”, where no SAW device was introduced; “With SAW” (prevention of biofilm growth), in which the SAW device was activated at the start of the experiment to inhibit biofilm development; and “With SAW” (removing biofilm), where biofilms were first allowed to form on glass slides before SAW activation to assess its biofilm-removal capability. Three freshwater microalgae species were selected to evaluate both biofilm prevention and removal: *Raphidocellis subcapitata* strain CS-327, a planktonic green alga, and two benthic diatom strains, CS-1664 and CS-1665. The experiments were conducted over varying durations—from several days to one month—to assess the sustained performance and longevity of the SAW device over time.

### 2.3. Experimental Setup of SAW Device for Preventing Algae Biofilm Formation

To propagate the SAW vibration, a Rigol function generator (RIGOL, DG2102, 100 MHz, NSW, Australia) was used to produce a radio frequency (RF) signal at the SAW device’s frequency. This signal was then sent to an amplifier (ZHL-5W-1+, Linear Amplifier, 5 MHz to 500 MHz, Mini-Circuits, Brooklyn, NY, USA) to boost the SAW power before transmitting it through an RF SMA cable to the SAW device. A DC power supply (TENMA, 72-2930, Element 14, Sydney, NSW, Australia) provided 24 V and 3 A to power the amplifier. In this experimental setup, the signal generator supplied a 120 Vpp excitation with a pulse duration of 70 ms, corresponding to an estimated power of 2.4 W, and the device was operated at an RF frequency of 16 MHz. The schematic diagram of the electrical connections for the SAW anti-biofouling setup is shown in Figure 3e, illustrating the amplified voltage being transmitted from the signal generator to the amplifier. A customized PCB (Figure 3d) was fabricated to enable automated switching between SAW-based anti-biofouling operation and turbidity measurements within a predefined periodic cycle. To direct the SAW wave toward the algal growth, a three-dimensional (3D) printed holder was fabricated to secure the SAW device and a glass slide representing the window of an optical sensor, which was connected to the amplifier via an RF SMA cable. The signal generator delivered a periodic power output with a 70 ms pulse occurring within a one-second interval, and the device’s RF frequency was appropriately set to operate the SAW device. The input voltage or power for a 16 MHz SAW device will vary with the amount of media, the type of algae, and the surface area to be protected against biofouling.

## 3. Result and Discussion

SAWs are widely utilized for manipulating microfluidics in numerous applications. In this study, we explore a novel potential use of the 16 MHz SAW device for preventing biofouling development on the surfaces of submerged water quality sensors, which has significant implications for the water industry and water quality monitoring systems by significantly lowering maintenance costs and improving the quality of water parameter measurements. The miniaturized 16 MHz SAW devices have the potential to control the biofouling growth on small windows of in situ optical sensors over an extended duration, which will lower maintenance costs for these sensors and improve the water quality monitoring system.

### 3.1. Experiment 1

**Figure 5:**
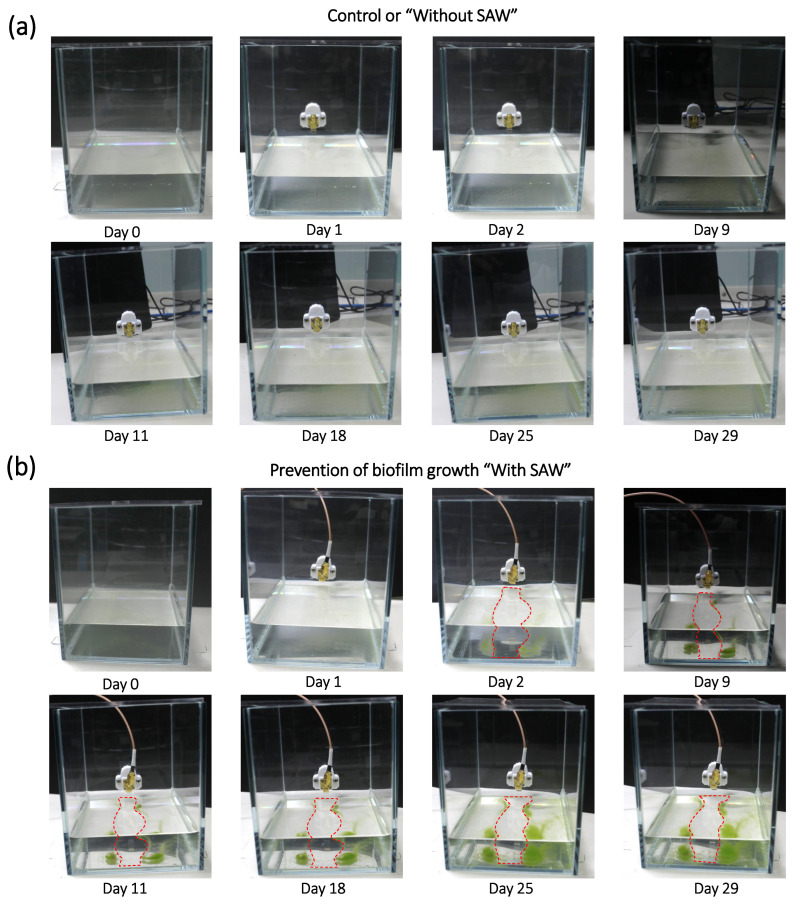
RGB images of algae tanks integrated with a 16 MHz SAW device illustrate the time-dependent inhibition of biofilm formation. (**a**) The control or “Without SAW” tank serves as a comparison, showing an almost uniform distribution of algae everywhere, and the media appear light green because of the algae. (**b**) The tank of “With SAW” shows displacement of algae or biofilm growth outside the active SAW vibrational area and the protected area (3.8 cm^2^) outlined in red, indicating the region effectively shielded by acoustic irradiation. Following approximately one day of algal cultivation, a 120 Vpp of electrical power with 70 ms duty cycle voltage was applied to the SAW device from Day 1. Both tanks were monitored in a one-month period, demonstrating the potential of SAW devices for long-term prevention of surface biofouling.

The objective of this experiment was to evaluate the ability of SAWs to prevent algal growth along a defined pathway at the bottom of glass tanks and to compare the results with control tanks operated “Without SAW” excitation. In this study, cultured planktonic *Raphidocelis subcapitata*, CS-327 algae, were introduced into 400 mL of a combination of tap water and algae media to facilitate algae growth. The algae were allowed to settle in the media for one day prior to activating the 16 MHz SAW device. A SAW device operating at a frequency of 16 MHz, with an amplitude of 120 Vpp and a duty cycle of 70 ms, was employed to evaluate its capacity to inhibit the accumulation and growth of algae and biofilm at the bottom of a glass tank measuring 15 × 11 × 13 cm. Over time, a distinct cleaning path, indicated by the red dotted line detected from biofilm growth, became apparent in Figure 5b. The leaky waves generated by the SAW were observed to displace the algae away from the vibrational region due to the active vibrational area and the progressive displacement of algae and biofilm from the surface in a month, as illustrated in Figure 5b. Conversely, the absence of SAW comparison, depicted in Figure 5a, resulted in increased algae growth within the tank, rendering the media greener. The images in Figure 5 were captured using a standard RGB camera. The comparison between the “With SAW” and “Without SAW” tanks demonstrates the effectiveness of SAW vibrational activity in preventing and protecting against algae proliferation for a small window.

### 3.2. Experiment 2

This experiment was designed to incorporate an interchangeable glass slide to capture the SAW-induced removal pathway and to elucidate the mechanism by which SAW propagation dislodges algal cells from substrate surfaces. A standard microscope slide (75 mm × 25 mm × 1 mm) was utilized to serve as the window of an optical sensor. Additionally, a 3D printed holder was printed using PETG filament material, with dimensions of 88 mm × 35 mm × 49 mm. The optimal distance was determined to be 1.25 mm × 1.25 mm, based on considerations of glass sliding flexibility and the proximity of the SAW device in relation to the glass slide, as illustrated in Figure 4a, for achieving optimal SAW vibration. The SAW device and glass slide are positioned perpendicular to each other at the edge to show the prevention of biofouling growth on their surfaces via a 16 MHz SAW device. Figure 4b illustrates a complete microscopic scan of the slide, taken with a 3D electronic microscope (Olympus, LEXT OLS5100, Notting Hill, VIC, Australia), highlighting the biofilm removal area in white in the long slide’s center. At the same time, the edges still show algae and biofilm. This study was conducted using strain CS-327 present in the medium and was performed under the same experimental parameters as Experiment 1. This experiment demonstrates the extent to which the region is kept free from algae and biofilm growth via SAW devices. The acoustic wave dislocates the micron-sized bio-organism due to its strong acoustic forces and keeps the surrounding surfaces free of algal growth. Figure 4c shows how the acoustic waves push algae away, preventing them from growing on the window surfaces of the in situ sensor. To better demonstrate how the 16 MHz SAW device produces these waves, we used a 6 μm red polystyrene particle (PRoSciTech, Kirwan, QLD, Australia). This particle size provides strong visual contrast and helps illustrate how acoustic forces move particles, similar to the algae in our experiment. The RGB image indicates that the mechanical waves push particles forward, keeping nearby surfaces clear and causing algae to settle only outside the SAW vibration area.

**Figure 6:**
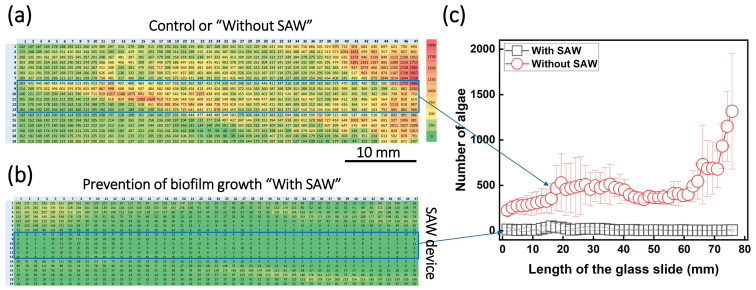
(**a**) The quantitative image of the glass slide control or “Without SAW” illustrates biofilm formation on its surfaces and the number of algae in each cell of the glass slide, while (**b**) depicts the glass slide treated “With SAW” to inhibit biofouling growth on its active SAW vibration area. (**c**) Comparison of the highlighted row, representing the potential sensor window area, for glass slides “With SAW” (☐) and “Without SAW” (◯), showing the difference in algal growth under SAW excitation. The comparison of algal abundance across corresponding sections of the two slides highlights the effectiveness of SAW vibrations in preventing and removing biofilm accumulation on surfaces in front of a submerged water-quality sensor window over time. Data are presented as mean ± standard deviation (n = 3).

Quantitative measurement of algae adhesion was performed using bright-field and fluorescent images. Images were captured with a PerkinElmer Operetta system equipped with a 10X LWD objective in fluorescence mode, using filter excitation at 410–430 nm and emission at 650–760 nm. A total of 893 fluorescence images per slide (arranged in a 47 × 19 grid) were analyzed with PerkinElmer Harmony 4.1 software to identify algae, exclude debris, and count algae. Each pixel on the slide indicates the level of algae presence in samples treated “With SAW” and “Without SAW”, as shown in Figure 6a,b with the SAW device positioned at the right-hand side of the slide. Green indicates fewer algae, while red shows more. The reduction and elimination of algae and biofilm growth due to strong SAW vibration is evident in Figure 6b, where algae counts decrease in the active vibration zone. Conversely, the “Without SAW” slide shows higher algae counts across the entire surface. A comparison of the middle rows of the “Without SAW” and “With SAW” slides in Figure 6c illustrates the effectiveness of SAWs in preventing and removing algae adhesion in that area. The “With SAW” sample shows minimal algal adherence, corresponding to an approximate prevention rate of 98%, attributable to the strong SAW-induced forces. The region selected for subsequent quantitative analysis spans approximately 70 mm in length and 10 mm in width and is highlighted by the blue rectangle in Figure 6a,b, attributable to the strong SAW-induced forces. The region selected for subsequent quantitative analysis spans approximately 70 mm in length and 10 mm in width and is highlighted by the blue rectangle in Figure 6a,b.

### 3.3. Experiment 3

**Figure 7:**
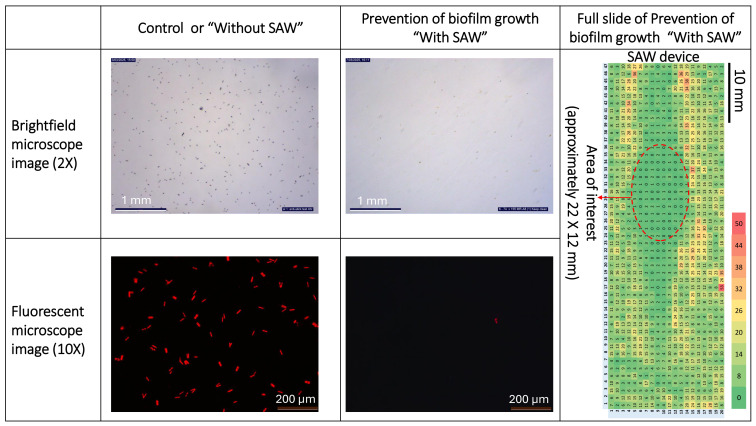
Microscopic images of glass slides labeled “Without SAW” and “With SAW” showing the presence and distribution of diatom CS-1664 algae. The first column presents 2X bright-field and 10X fluorescent images from the “Without SAW” slide, highlighting significant algal attachment in the absence of anti-biofouling treatment. In contrast, the second column displays images from the “With SAW” slide, demonstrating a notable reduction in biofouling due to the SAW device. The third column further illustrates the spatial variation in algal distribution across different regions of the “With SAW” slide, with the central red area showing the greatest anti-biofouling effect, attributed to strong acoustic vibrations. Quantitative analysis reveals approximately 127 algae within a 200 μm region on the “Without SAW” slide, while the “With SAW” slide contains only 2 algae in the same area. The magnified fluorescent images (10X) were captured within the defined area of interest, which corresponds to dimensions relevant to certain optical water-quality sensors. For this specific study, we considered the window of an optical turbidity sensor, and the window diameter is 15 mm.

A different type of algae, distinguished by its adhesiveness, was tested for this application. In this section, an adhesive diatom from inland waters (CS-1664) was examined to evaluate the prevention capabilities of the SAW as an anti-biofouling device. Figure 7 illustrates the surfaces labeled “With SAW” and “Without SAW,” highlighting the effectiveness of SAW acoustic waves in inhibiting diatom and biofilm growth in proximity to the activated area. The 2X bright-field and 10X fluorescent images reveal the algae morphology, which is rod-shaped and adheres to the glass slides in both “Without SAW” and “With SAW”. The magnified fluorescent (10X) images of both treatment conditions demonstrate the prevention of adhesive algae growth on the surfaces, attributed to a 120 Vpp voltage at the device’s RF frequency, which operated at a 70 ms of duty cycle for 22 h to keep the surfaces clean. A full glass slide labeled “With SAW” in the third column illustrates the amount of algae and biofilm in the area of interest, which could be any water-quality sensor’s window. This SAW device can assist underwater water-quality sensors in maintaining a clean window. Therefore, in future, we aim to attach this SAW device next to a window of an optical turbidity sensor (NTU, model-0001, In-situ Mare Optics, Bibra Lake, Australia), whose window diameter is 15 mm, to prevent it from algae and biofilm growth.

### 3.4. Experiment 4

**Figure 8:**
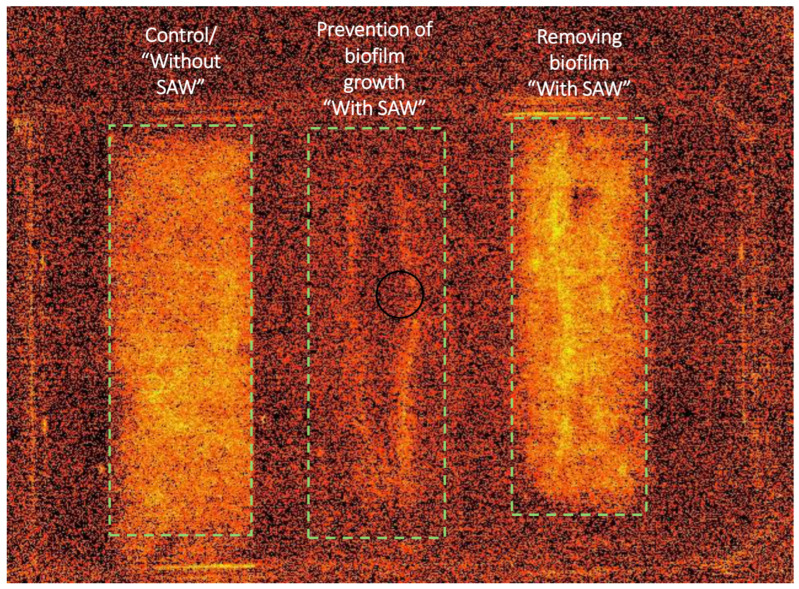
The viability and persistence of the diatom strain CS-1664 were assessed using an iPAM. The color gradient from orange to black reflects the relative abundance of viable algae on the slides, with brighter coloration indicating higher biomass. The slide labeled “With SAW” exhibits the lowest algal presence, demonstrating that 16 MHz SAW activation effectively dislodges algae through mechanical vibration. In contrast, the control or “Without SAW” slide shows the highest density of viable algae in bright orange color. The removing biofilm “With SAW” slide highlights potential biofilm removal occurring at the top region of the glass slide under the same SAW parameters. The black circle in the prevention biofilm growth “With SAW” image marks the iPAM imaging focal point, and the green dotted lines denote the boundaries of the glass slides.

To further evaluate the influence of the acoustic field effects algal growth, an imaging Pulse Amplitude Modulation fluorometry (iPAM; Walz Imaging PAM, IMAG-MAX/L, Heinz Walz GmbH, 91090 Effeltrich, Germany) was used to assess algal viability by quantifying photosynthetic activity. Glass slides were exposed to a 120 Vpp SAW input with a 70 ms duty cycle for 22 h, then transferred to a Petri dish containing fresh media for imaging. Figure 8 presents the viability of algae under three conditions: control or “Without SAW”, prevention biofilm “With SAW” and removing biofilm “With SAW”. Viable algae appear bright due to active photosynthesis, whereas dead, damaged, or absent algae appear dark or black color in the images. In the prevention biofilm growth “With SAW” slide, the central region of the slide shows minimal algal presence, while the control or “Without SAW” and removing biofilm “With SAW” slides display a greater abundance of algae. The removing biofilm “With SAW” slide shows a little biofilm disruption, particularly near the top of the slide. Together, these comparisons illustrate the differential effects of SAW activation on algal attachment and biofilm integrity. When interpreted alongside Figure 7, the results indicate that the 16 MHz SAW devices primarily displace algae and biofilm from the surface without compromising algal viability. It should be noted that the physiological condition of detached algal cells was not quantitatively assessed in the present study; targeted post-detachment viability analyses will be addressed in future work.

### 3.5. Experiment 5

**Figure 9:**
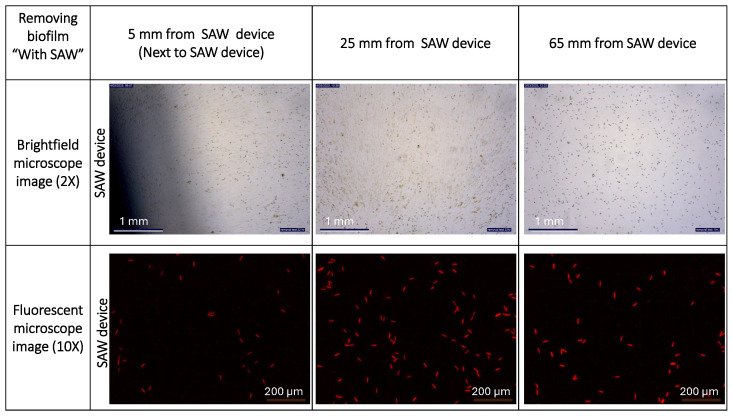
Microscopic images of removing biofilm “With SAW” slide—bright-field of diatom CS-1665 at 2X at the top and fluorescence at 10X at the bottom—show different regions of the slides treated “With SAW”. The bright-field images taken at 5 mm and 25 mm from the SAW device clearly illustrate the algae removal due to the SAW vibration, with a noticeable reduction in biofilm density near the active vibration zone. In contrast, less impact is observed at greater distances, such as 65 mm from the device. To be noted, a black shadow from the SAW device appears in the first bright-field image because it was positioned next to the glass slide. The fluorescence images (10X) display the rod-shaped algae and their quantity after 22 h of treatment at same parameters described earlier.

In Figure 9, we additionally examined the potential for cleaning biofilm induced by algae and other microorganisms. The thick biofilm developed over a span of four days under algal growth, while maintaining temperature and light conditions, inside a controlled temperature chamber within the Australian National Algae Culture Collection (ANACC) PC2/BC2 laboratory. Afterwards, the thick biofilm-containing glass slides were carefully placed onto the 3D glass holder in 400 mL of media. A 16 MHz SAW device was positioned vertically adjacent to a glass slide, as illustrated in Figure 4a, operating at 120 Vpp voltage at the respective device RF frequency, and a 70 ms duty cycle for a duration of 22 h to the glass slides to facilitate the biofilm removal process. The 2X bright-field and 10X fluorescent images demonstrate the potential for biofilm removal from the glass slide. The bright-field images distinctly illustrate the impact of the SAW device, dislodging the biofilm that was present at the commencement of the experiment, with some preventing action occurring at the midpoint of the slide. A smaller effect is observed at the edges and other parts of the glass slide, presumably due to the distance from the SAW vibration and the strong adhesion of the biofilm. We assume that extending the duration of this experiment may enhance the capability of the SAW device to eliminate the biofilm from the glass slide, with the required time for removal varying according to the thickness of the biofilm.

The observations described above were extended to another adhesive diatom species, CS-1665, to evaluate both biofilm prevention and the removal of mature biofilm, as presented in Figure S1. The experimental conditions remained consistent with previous trials, and the results clearly demonstrate the effectiveness of the 16 MHz SAW device in both preventing biofouling and removing established biofilm, without adversely impacting algal viability on the glass surfaces. Additionally, Figure S2 illustrates the use of a mixed culture of two adhesive diatoms, CS-1664 and CS-1665, to further elucidate the influence of SAW-induced vibrations on the removal of dense biofilms.

The experimental results obtained from this study suggest that the 16 MHz SAW device will serve as a promising candidate to shield in situ water quality sensors from underwater biofouling attacks for an extended duration, thereby potentially reducing the overall maintenance costs associated with the maintenance of total water quality monitoring systems. Additionally, this miniature device can be employed in various environments, including marine applications. Furthermore, its compact design and miniature size facilitate seamless integration with other water quality sensors, protecting them from biofilm development while ensuring that measurement integrity remains uncompromised.

The miniaturized 16 MHz SAW device can be positioned adjacent to an in situ sensor’s optical window to provide periodic, contactless cleaning at lower power consumption than commercially available mechanical brush systems. In this study, the estimated operating power of the developed SAW-based anti-biofouling device was approximately 2.4 W, compared with 4.2 W typically required for periodic operation of commercial mechanical brush systems used to clean optical sensor windows (NTU, model-0001, In-situ Mare Optics, Australia). Because the SAW device operates as a non-contact add-on, it eliminates the risk of surface abrasion or mechanical interference with the sensor window—a limitation commonly associated with brush-based cleaning mechanisms and particularly problematic for compact in situ sensors (as summarized in Table 1). Consequently, integration of the proposed SAW-based cleaning approach offers a practical solution to these constraints and has the potential to reduce maintenance demands, power consumption, and overall operational costs for long-term water quality monitoring networks.

### 3.6. Conclusions

To enhance and reduce the cost of the water quality monitoring system in Australia, we have developed a SAW-based anti-biofouling add-on device. This device is capable of working to prevent algae and biofilm growth on the windows of in situ optical water quality sensors, which reduces the total operational cost of the monitoring system. This device utilizes a combination of acoustic radiation force, centrifugal forces, and SAW physical vibration generated from 16 MHz SAW devices to displace algae, thereby restricting their growth in designated areas without interfering with the growth of microorganisms in the waterbodies.

Across seven experiments, we demonstrated the effectiveness of a 16 MHz SAW device in preventing biofouling growth on surfaces, as shown in the images and figures presented in the Section 3. This study examined different types of distinct algae: a planktonic *Raphidocelis subcapitata* CS-327 and two adhesive diatom strains, CS-1665, and CS-1664. The vertical propagation of SAW vibrations displaced the algae due to their acoustic radiation forces and SAW vibrations. It also contributed to the accumulation of algae outside the designated area of interest, shown in Experiment 1. As a result of the combination of acoustic forces, the 16 MHz SAW device was able to prevent algae growth approximately 98% across various types of media, shown in Experiments 2 and 3. We also examined the effect of high-frequency SAW mechanical vibration on algae viability using the iPAM facility. The distribution of live and dead diatoms observed in Figure 8 after 22 h of periodic SAW excitation confirms that SAW vibrations do not inhibit algal growth. Instead, as shown in Experiment 1, they help displace the algae, preventing biofilm formation on specific areas of surfaces. Furthermore, this study highlights the potential for biofilm removal when needed. By altering the power settings of the SAW device, the rate of surface cleaning can also be controlled. Experiments 6 and 7 in the Supplementary Information also demonstrated the effectiveness of preventing and removing biofilm growth on the CS-1665 diatom. This compact system can effectively prevent and remove biofouling from the sensor optical window without harming the surrounding ecosystem or interfering with data collection.

The 16 MHz SAW device has the potential to be employed for a variety of applications. They can be fabricated according to the specific area that requires the prevention or removal of algae and biofilm, which represents the principal advantage of these miniaturized devices. The subsequent phase of this project will involve integrating the miniaturized SAW device with commercially available in situ water quality sensors to safeguard them against biofouling attacks and to enhance the longevity of the sensors without compromising the measurement accuracy. The future trajectory of this research can be applied to the maintenance of submerged sensors in diverse environments, including marine and coastal water bodies. Due to its compact size and low power consumption, this device can also be modified to ensure the cleanliness of other in situ optical water quality sensors. Another future direction involves embedding the device directly into underwater in situ sensor housings, although this will require substantial engineering development. Key challenges include ensuring effective placement of the SAW device so that it prevents biofouling while avoiding interference with sensor measurements.

A limitation of this study is the relatively small surface area over which biofilm growth is actively suppressed, which may restrict immediate applicability to large-scale or industrial sensing systems. This constraint is primarily related to the selected SAW operating frequency and device geometry. Accordingly, future work will focus on systematic exploration of SAW frequency ranges and scalable device designs to extend the effective treatment area. Within the experimentally validated timeframe, the primary objective of this work was to demonstrate localized biofilm inhibition and the feasibility of removing mature biofilms from the optical windows of in situ water quality sensors, thereby reducing maintenance requirements and associated costs. Power consumption during continuous operation—particularly for miniaturized implementations—also remains an important consideration. Further optimization of power efficiency while maintaining effective anti-biofouling performance will be essential for long-term and autonomous deployments. The transparent lithium niobate substrate, functioning as a SAW delay line, enables compatibility with optical sensing platforms; however, careful device placement and angular alignment are required to minimize potential interference with optical measurements. These considerations highlight key directions for future system optimization and practical deployment.

## Figures and Tables

**Table 1:** Different methods of preventing biofouling on water quality sensors to extend sensor lifespan.

Methods	Mechanism	Advantages	Disadvantages	Applications	References
Biocide-Releasing Coatings	- Slowly release antimicrobial agents to kill microbial growth - Use complex compounds (e.g., copper, silver, Irgarol 1051, zinc pyrithione, tributyltin (TBT), copper)	- Long-term fouling control (6 months–3 years) - Proven history - Effective in marine settings - TBT are generally successful over time - Adapted by ∼70% of the global fleet (International Maritime Organization (IMO))	- Environmental risks due to leaching toxic compounds - Potential interference with sensor readings - TBT-based coating paints have been banned since 2008	- Sensor windows - Membranes - Marine applications	[5,11,12,13,14,15,16,17,18,19,20,21]
Anti-Adhesive Coatings	- To prevent the initial biofilm formation on the surfaces - Various types of polymers utilized, including super-hydrophobic polymers, slippery liquid-infused porous surfaces (SLIPS), protein or glycoprotein-based coatings, hydrophilic polymers, and zwitterionic polymers	- Non-toxic - Lower environment impacts than biocide coatings - No chemical residue	- Low stability - Provoking an immune response in some circumstance - Not suitable for long-term applications (3 h only) - Not biodegradable - High cost	- Biosensors - Biomedical implements - Food industries - Ship Hulls	[22,23,24,25,26,27,28,29,30,31]
Mechanical Methods (Wipers/Brushes)	- Mechanical forces to eliminate biofilm from surfaces - Prevent biofouling establishment	- One of the most straightforward methods - No chemical residue - Environmental friendly options available	- Less effective for application with sensitive equipment - Can sometimes be rigid [11] - Additional cost and higher power demands	- In situ optical and non-optical water quality sensors	[32,33,34,35,36,37,38]
Ultrasonic Waves	- Operate below 20 kHz to prevent microorganisms - Periodic emissions of high-power ultrasonic waves	- Environmental friendly and non-toxic - Can mitigate excessive fuel consumption if deployed on ship halls	- Potential high cost, (prices ranging from EUR 1200 to 7000) - Less suitable for small surfaces, such as a water quality sensor windows due their small size	- Generally used for motorboats, sailboats, and various surfaces - Vessel hulls	[7,39,40,41,42,43,44]

**Table 2:** The summary of all experiments conducted on a 16 MHz SAW device used for anti-biofouling, testing various diatom types and conditions, where “◯” shows that the experiment has been conducted, and “*X*” shows that the experiment has not been conducted. Different methods of preventing biofouling on water quality sensors to extend sensor lifespan.

Experiment No.	Type of Algae	Control or “Without SAW”	Prevention of Biofilm Growth “With SAW”	Removing Biofilm “With SAW”	Duration of the Experiment
**Experiment 1**	CS-327	◯	*X*	◯	29 days
**Experiment 2**	CS-327	◯	◯	*X*	29 days
**Experiment 3**	CS-1664	◯	◯	*X*	22 h
**Experiment 4**	CS-1664	◯	◯	◯	22 h
**Experiment 5**	CS-1664	◯	*X*	◯	22 h
**Experiment 6**	CS-1665	◯	◯	◯	22 h
**Experiment 7**	CS-1664 + CS-1665	◯	*X*	◯	22 h

## Data Availability

The original contributions presented in this study are included in the article/Supplementary Material. Further inquiries can be directed to the corresponding author.

## Supplementary Materials

The following supporting information can be downloaded at: https://www.mdpi.com/article/10.3390/s26113480/s1, Figure S1: Fluorescent microspic and iPAM images of CS-1665 after 22 hours of treatment; Figure S2: Fluorescent microspic images of a mixture of CS-1664 and CS-1665 after 22 hours of treatment; Video S1: A video of displacing targets via a 16 MHz SAW device.
