# Supplementary material for: Exploring Surface Acoustic Waves (SAWs) for Water Quality Sensor’s Anti-Biofouling Application: A New Direction for Acoustic Waves
Source: Sensors (Basel). 2026 Jun 1;26(11):3480. doi: 10.3390/s26113480 (PMC13259138; doi:10.3390/s26113480)
Supplement: Supplementary file 1 [file sensors-26-03480-s001.zip › sensors-4250756-supplementary-english.pdf]

Supplementary Information

Exploring Surface Acoustic Waves (SAWs) for Water Quality Sensor's Anti-biofouling Application: A New Direction for Acoustic Waves

This supplementary information document includes: a video demonstrating how the 16 MHz surface acoustic waves (SAW) displaces the algae and biofilm growth from the activated area via SAW wave propagation; (Experiment 6) microscopic images of the adhesive diatom CS-1665 used to illustrate the impact of SAW activity; and (Experiment 7) results from experiments using a combination of two adhesive diatoms, CS-1664 and CS-1665, to demonstrate both the prevention of biofilm formation and the removal of existing biofilm from surfaces.

Experiment 6:

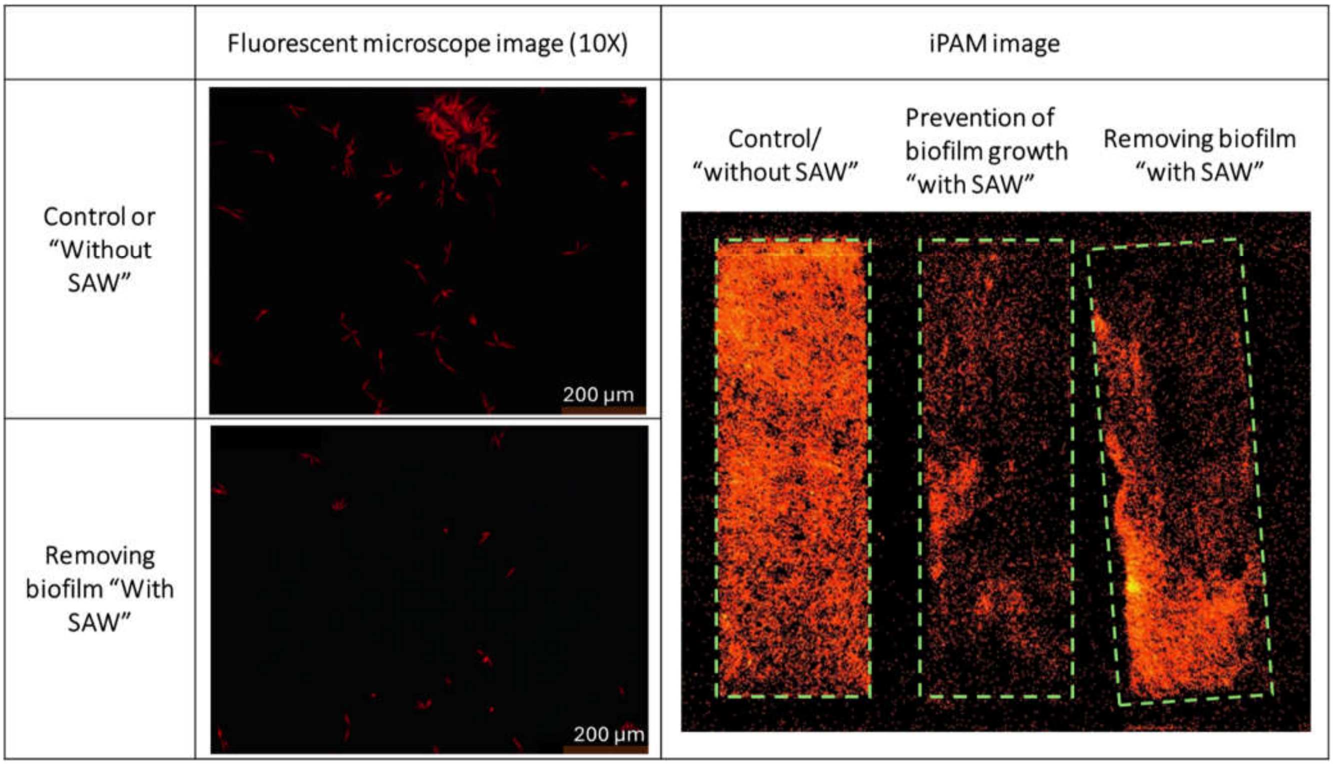

Figure S1: The first column presents 10X fluorescent microscopic images of CS-1665 diatoms on glass slides labeled Control or "Without SAW" and Prevention of biofilm growth "With SAW," illustrating the effect of SAW on removing adhesive diatom biofilm. The biofilm was grown in a controlled environment (ANACC PC2/BC2 Laboratory) for 4 days, then transferred to 400 mL of media in glass tanks for the experiment. The second column shows iPAM images comparing the viability of CS-1665 under three conditions: control or "Without SAW", prevention of biofilm growth "With SAW", and removing biofilm "With SAW" after 22 hours of periodic SAW treatment at 120 Vpp of a 70 ms duty cycle and 16 MHz resonance frequency. Green dotted lines are used to indicate the edges of the different treated glass slides within a single Petri dish.

Experiment 7:

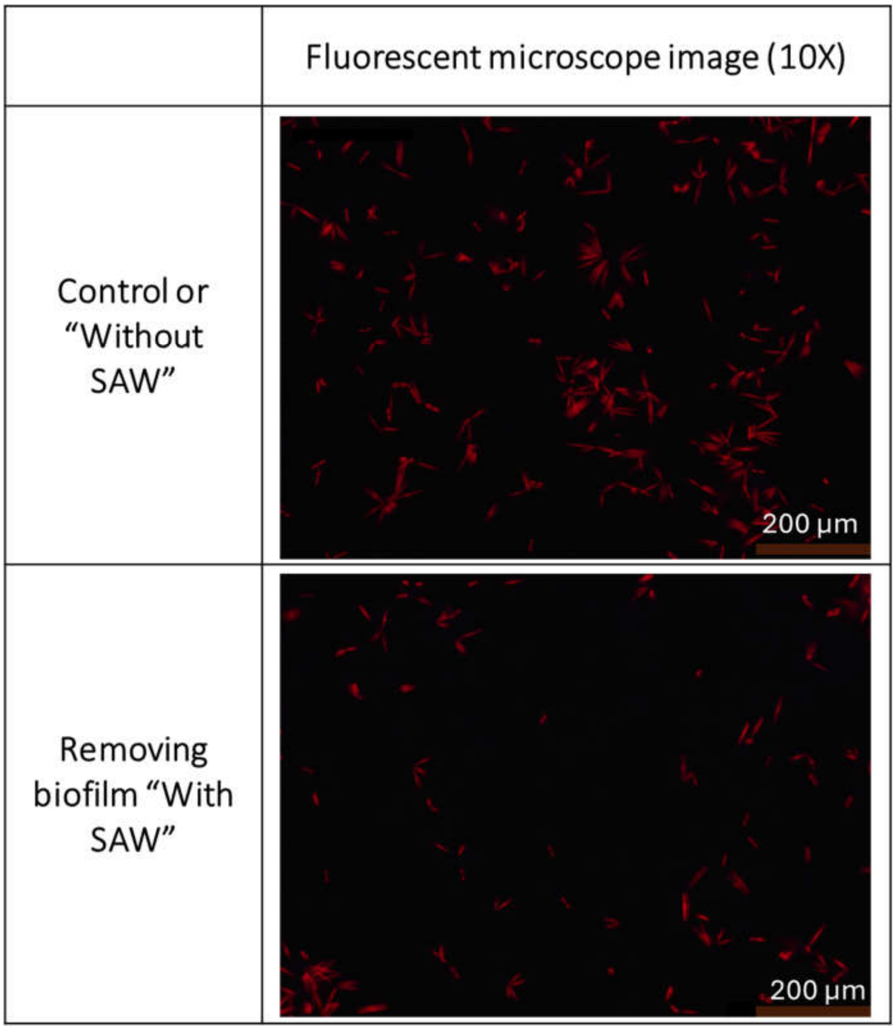

Figure S2: A mixture of adhesive diatom strains CS-1664 and CS-1665 was cultured on microscope glass slides for 4 days to allow the formation of a thick biofilm. The thick biofilm-coated slides were then placed in a 3D-printed holder and submerged in 400 mL of growth media for experimentation. Fluorescent microscopic images of the slides labeled "Without SAW" and "With SAW" illustrate the potential for removing thick biofilm from surfaces after 22 hours of periodic SAW treatment.
